# TdP Incidence in Methoxamine-Sensitized Rabbit Model Is Reduced With Age but Not Influenced by Hypercholesterolemia

**DOI:** 10.3389/fphys.2021.692921

**Published:** 2021-06-21

**Authors:** Lukáš Nalos, Dagmar Jarkovská, Jitka Švíglerová, Annabell Süß, Jakub Záleský, Daniel Rajdl, Milada Krejčová, Jitka Kuncová, Josef Rosenberg, Milan Štengl

**Affiliations:** ^1^Department of Physiology, Faculty of Medicine in Pilsen, Charles University, Pilsen, Czechia; ^2^Biomedical Center, Faculty of Medicine in Pilsen, Charles University, Pilsen, Czechia; ^3^Institute of Clinical Biochemistry and Haematology, Faculty of Medicine in Pilsen, Charles University, Pilsen, Czechia; ^4^New Technologies for the Information Society, Faculty of Applied Sciences, University of West Bohemia, Pilsen, Czechia

**Keywords:** hypercholesterolemia, TdP arrhythmia, arrhythmia risk prediction, rabbit, amplitude-aware permutation entropy

## Abstract

Metabolic syndrome is associated with hypercholesterolemia, cardiac remodeling, and increased susceptibility to ventricular arrhythmias. Effects of diet-induced hypercholesterolemia on susceptibility to torsades de pointes arrhythmias (TdP) together with potential indicators of arrhythmic risk were investigated in three experimental groups of Carlsson’s rabbit model: (1) young rabbits (YC, young control, age 12–16 weeks), older rabbits (AC, adult control, age 20–24 weeks), and older age-matched cholesterol-fed rabbits (CH, cholesterol, age 20–24 weeks). TdP was induced by α-adrenergic stimulation by methoxamine and I_Kr_ block in 83% of YC rabbits, 18% of AC rabbits, and 21% of CH rabbits. High incidence of TdP was associated with high incidence of single (SEB) and multiple ectopic beats (MEB), but the QTc prolongation and short-term variability (STV) were similar in all three groups. In TdP-susceptible rabbits, STV was significantly higher compared with arrhythmia-free rabbits but not with rabbits with other than TdP arrhythmias (SEB, MEB). Amplitude-aware permutation entropy analysis of baseline ECG could identify arrhythmia-resistant animals with high sensitivity and specificity. The data indicate that the TdP susceptibility in methoxamine-sensitized rabbits is affected by the age of rabbits but probably not by hypercholesterolemia. Entropy analysis could potentially stratify the arrhythmic risk and identify the low-risk individuals.

## Introduction

Torsades de pointes arrhythmia (TdP) is a potentially life-threatening polymorphic ventricular tachycardia arising on the basis of QT interval prolongation. QT interval prolongation is a manifestation of decreased repolarization reserve, which comprises a substrate for TdP arrhythmia (Roden, 1998). Deteriorated repolarization reserve is associated with several congenital syndromes; acquired conditions, such as ischemia or heart failure; and also a wide variety of medications (Antoniou et al., 2017). Many drugs were withdrawn from the market or their development was terminated because of their potential to induce TdP or prolong QT interval (Redfern et al., 2003; Valentin, 2010). Metabolic syndrome is associated with abnormal electrical activities in the myocardium characterized by prolonged QT interval in the ECG, QT interval dispersion, and ventricular arrhythmias (Soydinc et al., 2006; Li et al., 2009; Karaagac et al., 2014; Park and Lee, 2018). Hypercholesterolemia induces electrical remodeling of cardiomyocytes and sympathetic nerve sprouting leading to increased vulnerability of the heart to ventricular fibrillations (Chen et al., 2001; Liu et al., 2009). Hyperlipidemia has a direct prolonging effect on action potential duration (Liu et al., 2009) and QT/QTc interval (Szabó et al., 2005). Treatment with statins lowers cardiovascular risk by reduction of QTc dispersion and ventricular premature complexes (Gualdiero et al., 2002; Vedre et al., 2009) and prevents prolongation of action potential duration (Liu et al., 2009). Despite an apparent link between hypercholesterolemia, QT prolongation, and TdP, little is known about the effect of hypercholesterolemia on TdP arrhythmia inducibility. Most published results concerning hypercholesterolemia and arrhythmogenicity came from models combining high cholesterol and high triglyceride plasma levels, and arrhythmia models were limited to rapid pacing-induced ventricular fibrillations or arrhythmias due to myocardial ischemia/reperfusion injury. To investigate the effects of hypercholesterolemia on long-QT arrhythmia inducibility, the Carlsson’s methoxamine sensitized rabbit model of TdP arrhythmias (Carlsson et al., 1990) was combined with diet-induced hypercholesterolemia.

Age of the rabbits used for the Carlsson’s model of TdP arrhythmias is usually between 12 and 16 weeks as these animals are considered to be adult (de Turckheim et al., 1983). Because rabbits in our study were fed with a cholesterol diet for 8–12 weeks, an additional age-matched group was included resulting in three experimental groups: (1) young rabbits (aged 12–16 weeks, comparable to other published data), (2) cholesterol-fed rabbits (aged 20–24 weeks), and (3) age-matched rabbits (aged 20–24 weeks).

Despite enormous scientific effort, the prediction of (TdP) arrhythmia risk remains doubtful (Schwartz and Woosley, 2016). It became apparent that the simple prolongation of the QT interval is not a reliable indicator of arrhythmic risk (De Vecchis et al., 2018). Some spatial and temporal heterogeneity criteria, such as index of cardiac electrophysiological balance (Lu et al., 2013), prolongation of the interval between the peak and the end of the T wave (Tpeak to Tend) on the 12-lead ECG (Panikkath et al., 2011; Delinière et al., 2019), or beat-to-beat variability of repolarization (BVR) quantified as short-term variability (STV; Bossu et al., 2017) were recently introduced as potential risk indicators. Arrhythmia risk prediction by these methods, however, relies on analysis of the ECG signal preceding shortly the arrhythmic event and usually in the presence of pharmacological challenge. Therefore, in our study, we tried to enhance the predictive power by using entropy-based methods for analyzing baseline ECG prior to any challenge.

In general, analysis of entropy might reveal tiny irregularities of the ECG signal that perhaps occur in rabbits susceptible to TdP after pharmacological challenge. Abilities of permutation entropy (Bandt and Pompe, 2002) to capture the underlying dynamics of the time series are shown in a diversity of fields, including medicine (Olofsen et al., 2008). In our study, two recently introduced approaches for analysis of time series were used: (1) new empirical amplitude-aware permutation entropy (AAPE) was adopted to overcome the lack of amplitude consideration in standard permutation entropy analysis (Unakafova, 2015; Azami and Escudero, 2016; Cuesta-Frau et al., 2019); (2) information exergy index (IEIN) combines singular value decomposition and information exergy and allows capturing of the time development of irregularities (Zhang et al., 2013). These choices were based on comparative analysis of various techniques of time series analysis, such as complexity (Rosso et al., 2007), ergodicity, and mixing (Crutchfield and Feldman, 2003); detrended fluctuation analysis (Peng et al., 1994); and amplitude- and fluctuation-based dispersion entropy (Azami and Escudero, 2018).

## Materials and Methods

### Animals

The study was conducted according to the guidelines of the Declaration of Helsinki and approved by the Committee for Experiments on Animals of the Charles University Faculty of Medicine in Pilsen and by the Ministry of Education, Youth, and Sports of the Czech Republic (protocol number MSMT-47402/2012–30). Only female rabbits were used in order to avoid gender variability and because higher susceptibility to TdP arrhythmias was reported for both female patients and rabbits (e.g., Ebert et al., 1998). 37 female New Zealand white rabbits were divided into three groups: (1) young (juvenile) control rabbits fed standard chow (YC; *n* = 12; age 12–16 weeks, and mean weight 2.4 kg), (2) adult control rabbits fed standard chow (AC; *n* = 11; age 20–24 weeks, and mean weight 4.1 kg), and (3) adult hypercholesterolemic rabbits fed standard chow supplemented with 1% cholesterol for 8–12 weeks (CH; *n* = 14; age 20–24 weeks, and mean weight 4 kg). All rabbits were fed *ad libitum*. Content of cholesterol in standard chow was below 0.01%. Serum levels of total cholesterol, HDL cholesterol, and triglycerides were examined. Levels of total cholesterol, HDL-cholesterol, and triglycerides were measured in a clinical laboratory with routinely used enzymatic colorimetric methods by Roche on an automatic analyzer by Roche (Cobas c 8000, Basel, Switzerland). Shortly, total cholesterol was determined with the cholesterol oxidase method and HDL-cholesterol with cholesterol esterase and oxidase after masking non-HDL particles and triglycerides with a series of enzymatic reactions (lipoprotein lipase, glycerol kinase, and glycerolphosphate oxidase). Formation of a colored substance that is photometrically detected in all three methods is based on oxidation of a substrate with hydrogen peroxide; reaction is catalyzed by peroxidase. LDL cholesterol was calculated with the Friedewald equation (LDL = TC – HDL – TAG/2,2).

### *In vivo* Experiments

The methoxamine-sensitized rabbit model of TdP arrhythmia as described by Carlsson et al. (1990) was employed with minor modifications. Anesthesia was induced and maintained with ketamine (35 mg/kg) and xylazine (5 mg/kg) applied every 30 min i.m. The external jugular vein was exposed and cannulated for drug application. An ECG lead II was recorded using needle electrodes (Biopac System; Biopac Systems Inc, Goleta, CA, United States). After 10 min of recovery from surgery, control ECG was measured for 10 min. Then application of methoxamine (15 μg/kg/min, 2 ml/h) was started and continued for another 10 min. This was followed by application of lower-dose dofetilide (10 μg/kg/min, 2 ml/h) for 20 min on top of methoxamine. If no TdP arrhythmia developed, a higher dose of dofetilide (20 μg/kg/min, 4 ml/h) was administered. The experiment was terminated after 20 min on a higher dose of dofetilide or after 20 s of persistent TdP arrhythmia. In dofetilide groups, data from recordings at the higher dose of dofetilide were analyzed and reported unless otherwise stated.

### Solutions and Chemicals

Dofetilide was initially dissolved in 0.1 mM HCl (25 mg/ml) and diluted in 0.9% saline to the required concentration. Methoxamine HCl was dissolved and diluted in 0.9% saline. All chemicals were purchased from Sigma Aldrich.

### Calculations

ECG data were first analyzed manually in Biopac Student Lab 4.1 (Biopac System; Biopac Systems Inc, Goleta, CA, United States). The animals were considered TdP inducible when at least three TdP episodes were observed or when a TdP episode lasted more than 10 s. TdP was defined as a polymorphic ventricular tachyarrhythmia with at least five consecutive undulating QRS complexes with a typical twisting around the isoelectric line of the ECG. We analyzed single ectopic beats (SEB), multiple ectopic beats (MEB), and bigeminy. MEB was defined as series of two to four ectopic beats. In one animal, atrio-ventricular block occurred. For automatic ECG analysis, in-house made software was used. Heart rate-corrected QT values were calculated using the formula especially developed for this animal model (Carlsson et al., 1993). BVR was determined as a STV: 30 consecutive beats were used for the calculation

$$\mathrm{STV}=\sum \frac{\left|D_{n-1}-D_{n}\right|}{30\,\sqrt{2}},$$

where *D* represents QTc (Thomsen et al., 2004).

The heart rate variability (HRV) was analyzed in MATLAB 2014b (MathWorks Inc., Natick, MA, United States, 2014) in intervals of the last 5 min of the baseline recording. R peaks were detected by the derivative-threshold algorithm (Pan and Tompkins, 1985). Ectopic beats were determined automatically (difference more than 20% from the mean RR-interval) and replaced using linear interpolation. The Lomb–Scargle periodogram (Lomb, 1976; Scargle, 1982; Press and Rybicki, 1989) was applied to estimate the power spectral density. Power in the low (LF; 0.04–0.15 Hz) and high (HF; 0.15–0.40 Hz) frequency range were calculated.

For entropy analysis of the ECG signal, a Matlab code was developed (MATLAB R2017a; MathWorks Inc., Natick, MA, United States, 2014) using the algorithms for calculation of AAPE (Azami and Escudero, 2016) and IEIN (Zhang et al., 2014) based on robust empirical permutation entropy rePEnew (Unakafova, 2015). Analysis of each ECG record provided values of AAPE and IEIN σ (Tian et al., 2018). The first parameter characterizes the (ir)regularity of the ECG signal, whereas the second one describes its development in time. Sections of ECG records without artifacts were selected for analysis.

### Statistical Analysis

Statistical analysis was performed using Origin 2017 (OriginLab Corp., Northampton, MA, United States). Data are presented as the mean ± SD. Statistical comparisons were made with one- or two-way repeated-measures ANOVA followed by *post hoc* Tukey test where appropriate. Outliers were identified using Grubbs test. Incidence was tested with the two-sample proportion test. Differences at *p* ≤ 0.05 were considered to be significant.

## Results

**FIGURE 1:**
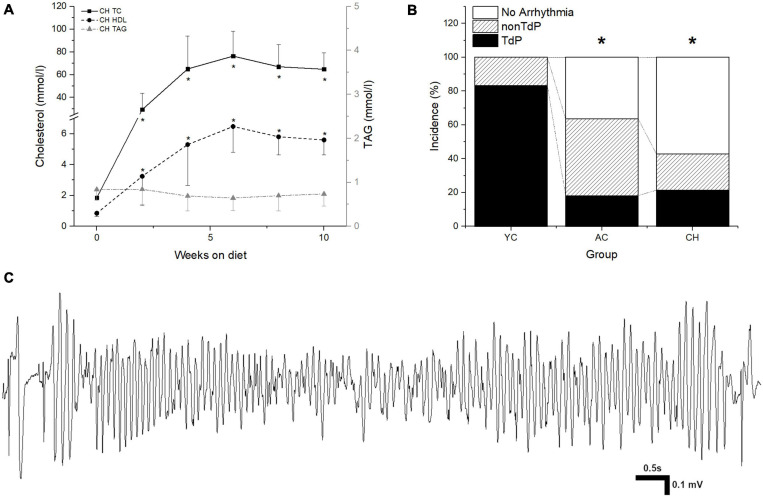
**(A)** Total cholesterol (TC), HDL cholesterol (HDL), and triacylglycerol (TAG) plasma levels in cholesterol-fed rabbits (CH). *p ≤ 0.05 vs. baseline. **(B)** Incidence of TdP and non-TdP arrhythmias in young rabbits (YC), adult control rabbits (AC), and cholesterol-fed rabbits (CH). *p ≤ 0.05 vs. YC. **(C)** Representative recording of TdP arrhythmia.

In CH, plasma levels of total cholesterol (baseline 1.85 ± 0.42 vs. 67 ± 19.6 mmol/L after 8 weeks of diet) as well as of HDL cholesterol (baseline 0.85 ± 0.2 vs. 5.8 ± 1.16 mmol/L after 8 weeks of diet) increased significantly, whereas the levels of triglycerides remained stable throughout the experiment (Figure 1A). LDL cholesterol was significantly increased in the CH group (baseline 0.57 ± 0.32 vs. 61 ± 22.3 mmol/L after 8 weeks of diet). In AC the plasma levels of TC, HDL, LDL, and TAG were comparable to those at baseline in CH group and did not change significantly throughout the experiment (total cholesterol: baseline 1.68 ± 0.47 vs. 1.65 ± 0.24 mmol/L after 8 weeks of normal diet, HDL cholesterol: 0.91 ± 0.12 vs. 1.1 ± 0.16 mmol/L, LDL cholesterol: 0.51 ± 0.3 vs. 0.39 ± 0.17 mmol/L, and triglycerides: 0.8 ± 0.3 vs. 0.36 ± 0.03 mmol/L).

In the YC group, TdP arrhythmia could be induced in 10 out of 12 animals in the presence of a higher dose of dofetilide (inducibility 83%, Figures 1B,C). In the AC group, TdP only occurred in 2 out of 11 animals in the presence of a higher dose of dofetilide (inducibility 18%, Figure 1B). In the CH group, TdP were observed in 3 out of 14 animals in the presence of a higher dose of dofetilide (inducibility 21%, Figure 1B). Besides TdP arrhythmias, other arrhythmic events (SEB and/or MEB, bigeminy) occurred in all three groups: YC 12/12, 100%; AC 7/11, 64%; and CH 6/14, 43% (Figure 1B).

**FIGURE 2:**
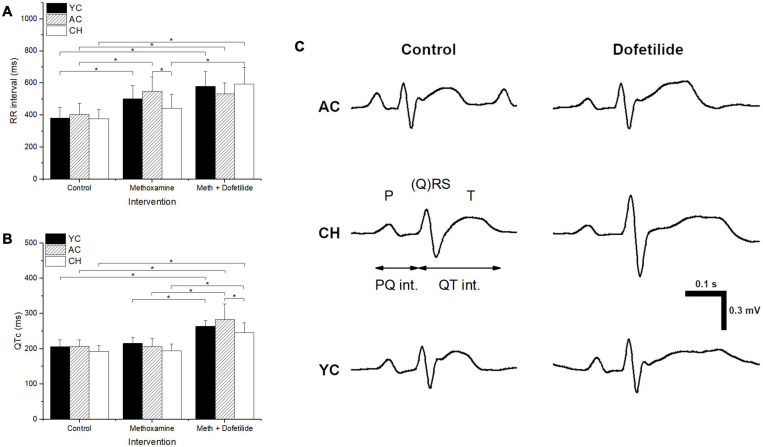
ECG characteristics of young rabbits (YC), adult control rabbits (AC), and cholesterol-fed rabbits (CH) at baseline, in the presence of methoxamine, and in the presence of methoxamine + dofetilide. *p ≤ 0.05. **(A)** RR interval, **(B)** QTc interval. **(C)** Representative examples of ECG.

Baseline ECG parameters were not significantly different between the groups (Table 1 and Figure 2C). During infusion of methoxamine, the RR interval was prolonged significantly in YC and AC, but reflex bradycardia was not significant in the CH group (Table 1 and Figure 2A). Other ECG intervals remained unchanged (Table 1), and no arrhythmias were observed. Application of dofetilide further increased RR intervals (significant to baseline) and induced significant dose-dependent prolongation of the QTc interval in all groups (Table 1 and Figure 2B).

**TABLE 1:** ECG parameters of the rabbits.

	**Control**			**Methoxamine**			**Methoxamine + Dofetilide**		
	**YC**	**AC**	**CH**	**YC**	**AC**	**CH**	**YC**	**AC**	**CH**
RR [msec]	381 ± 66	404 ± 69	376 ± 57	502 ± 81*	549 ± 88*	443 ± 83^$#^	579 ± 91*	532 ± 67*	593 ± 104^*#^
PQ [msec]	68 ± 4	73 ± 4	72 ± 5	73 ± 5^$#^	73 ± 3	74 ± 5	71 ± 5	83 ± 7^*#^	86 ± 22
QRS [msec]	68 ± 5	75 ± 6	74 ± 6	71 ± 5^$#^	76 ± 6	77 ± 8^$#^	78 ± 5	95 ± 19^*#^	87 ± 14
QT [msec]	220 ± 26	225 ± 26	205 ± 21	250 ± 24	250 ± 31	228 ± 43	312 ± 19^*#^	324 ± 46^*#^	297 ± 30^*#^
QTc [msec]	205 ± 19	207 ± 17	192 ± 17	215 ± 17	206 ± 23	203 ± 42	263 ± 15^*#^	283 ± 43^*#^	246 ± 27^*#^$#^^
STV [msec]	5.4 ± 2.2	5.1 ± 1.4	5.8 ± 2.6	6.6 ± 1.6	6 ± 1.5	6.2 ± 1.7	11.5 ± 12.3	8.5 ± 3.6	12.3 ± 10.4^*#^

YC, young control group; AC, adult control group; and CH, cholesterol fed group.*p ≤ 0.05 vs. Control, ^#^*p* ≤ 0.05 vs. Methoxamine, and ^$^*p* ≤ 0.05 vs. AC group.

**FIGURE 3:**
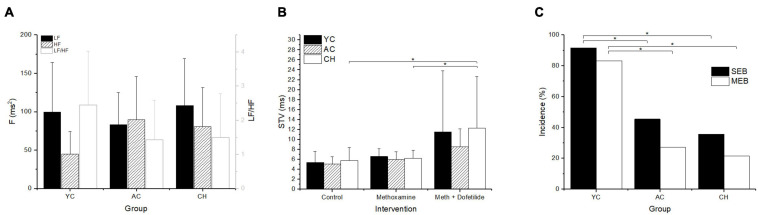
Autonomic drive, STV and ectopic activity in young (YC), adult control (AC), and cholesterol-fed rabbits (CH). **(A)** HRV expressed as power in the low (LF) and high (HF) frequency bands and ratio of LF/HF (LF/HF). **(B)** Short term variability (STV) of QTc. **(C)** Incidence of single (SEB) and multiple (MEB) ectopic beats. *p ≤ 0.05.

Because the differences in inducibility between young and adult rabbits could possibly be attributed to different autonomic nervous system drive, the frequency domain analysis of the HRV was performed to quantify changes in autonomic regulation. No significant difference between the groups were found for both high- and low-frequency bands (Figure 3A). STV of QTc interval (STV) was significantly increased in the presence of dofetilide only in the CH group (Figure 3B). Occurrences of SEB or MEB were manually evaluated in ECG recordings in the presence of dofetilide prior to TdP arrhythmia (Figure 3C). Incidence of SEB and MEB was significantly higher in the YC group compared with the AC and CH groups.

**FIGURE 4:**
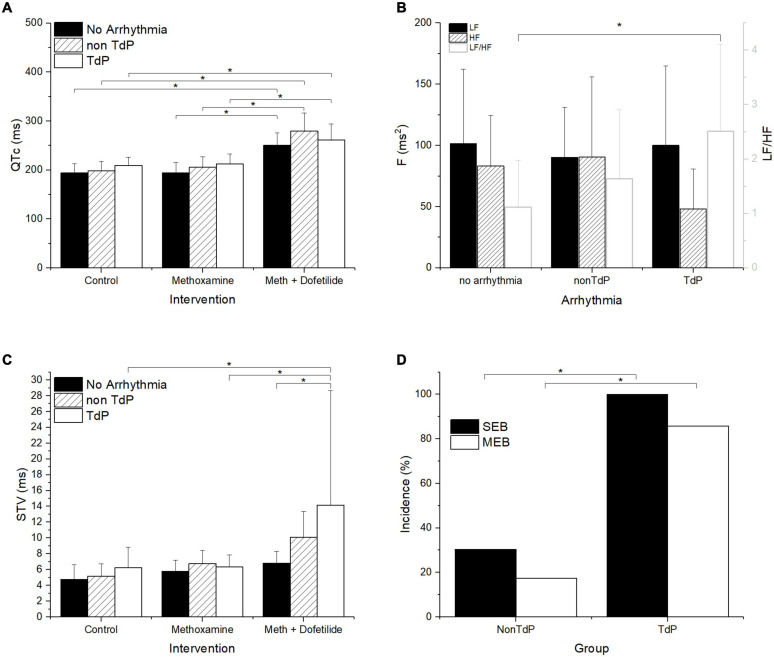
QTc, autonomic drive, STV and ectopic activity in rabbits without arrhythmia (no arrhythmia), with non-TdP arrhythmias (non TdP), and with TdP arrhythmia (TdP). **(A)** QTc interval, **(B)** HRV expressed as power in the low (LF) and high (HF) frequency bands and ratio of LF/HF (LF/HF). **(C)** Short term variability (STV) of QTc. **(D)** Incidence of single (SEB) and multiple (MEB) ectopic beats. *p ≤ 0.05.

In search of predictive indicators of TdP arrhythmia risk, the animals were regrouped into groups of TdP responders (*n* = 14), in which TdP arrhythmias developed, and non-responders without TdP arrhythmia (*n* = 23), which were further subdivided into the group of no arrhythmias (*n* = 12) without any arrhythmic events and a group of non-TdP arrhythmias (*n* = 11), in which arrhythmic events other than TdP (SEB, MEB, and bigeminy) were observed. In all three groups, QTc was significantly prolonged only in the presence of dofetilide, and there was no significant difference between the groups (Figure 4A). Analysis of HRV at baseline revealed a significantly lower HF component and consequently higher LF/HF ratio in the group of TdP responders when compared with the group of no arrhythmias (Figure 4B). STV increased significantly only in the group of TdP responders in the presence of dofetilide when it was significantly higher than in the group of no arrhythmias but not the group of non-TdP arrhythmias (Figure 4C). Incidence of SEB and MEB in the presence of dofetilide were significantly higher in the group of TdP responders (Figure 4D) providing TdP prediction sensitivity of 1 and 0.86 and specificity of 0.7 and 0.83 for SEB and MEB, respectively.

**FIGURE 5:**
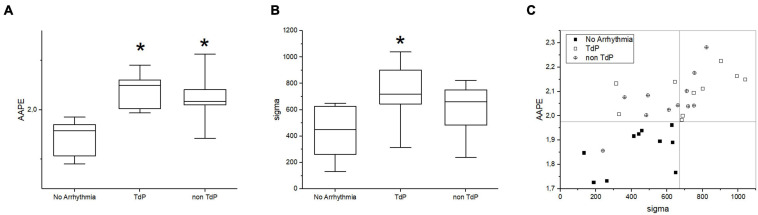
Entropy analysis in rabbits without arrhythmia (no arrhythmia), with non TdP arrhythmia (non TdP), and with TdP arrhythmia (TdP). **(A)** Amplitude-aware permutation entropy (AAPE), **(B)** information exergy index (IEIN, sigma). **(C)** AAPE and IEIN relation; arbitrary threshold 1.975 and 670, respectively. *p ≤ 0.05 vs. no arrhythmia group.

Not all recordings had enough long sections without artifacts for entropy analysis, so only ECG recordings of 31 rabbits could be analyzed. The sizes of groups were TdP responders, 10 rabbits; no arrhythmias group, 10 rabbits; and group of non-TdP arrhythmias, 11 rabbits. Analysis of entropy in baseline ECG recordings (without any pharmacological challenge on board) using AAPE and IEIN approaches revealed significantly lower entropy parameters in the group of no arrhythmias (Figures 5A,B). When both AAPE and IEIN were combined, the arrhythmia-free animals clustered in the bottom-left quadrant (Figure 5C). AAPE analysis allowed determination of low-risk (arrhythmia-free) animals with excellent sensitivity and specificity (sensitivity of 1, specificity of 0.91; arbitrary threshold 1.975), whereas IEIN showed lower specificity (sensitivity of 1, specificity of 0.53; arbitrary threshold 670).

## Discussion

In the first part of the study, the influences of age and hypercholesterolemia on the susceptibility to TdP arrhythmias in a methoxamine-sensitized rabbit model were investigated. In the second part of the study, several indicators of proarrhythmic risk were analyzed to determine their predictive power.

### Effect of Age on TdP Inducibility

In young rabbits (age 3–4 months), high susceptibility to TdP arrhythmias (TdP induced in 83% of young rabbits) comparable to earlier reports (e.g., Carlsson et al., 1990, 1993; Vincze et al., 2008) was observed, whereas in adult rabbits (age 5–6 months) the TdP inducibility was significantly lower regardless of plasma cholesterol levels (TdP induced in 18% of adult normocholesterolemic rabbits vs. 21% of adult hypercholesterolemic rabbits). Although the rabbits of age 3–4 months (weight of 2–3 kg) are often used in experimental research as adult animals, they should be rather considered peripubertal because puberty in rabbits occurs from 3 to 5 months of age (Kamwanja and Hauser, 1983; Laffan et al., 2018). Detailed ontogenetic studies focusing on cardiac development in rabbits within this age period (3–4 vs. 5–6 months) are missing; however, with regard to the peripubertal time window, effects of varying levels of sex hormones are likely. Progesterone and estrogens are reported to exert opposing effects on the heart. Progesterone pretreatment is shown to reduce the incidence of drug-induced TdP in atrioventricular node-ablated isolated perfused rabbit hearts (Tisdale et al., 2019) and to protect against prolongation of action potential duration – APD (90) and triangulation associated with potassium channel inhibition (Tisdale et al., 2011). In isolated guinea pig ventricular myocytes, progesterone shortened action potential duration, probably due to enhancement of the slow delayed rectifier K^+^ current (I_Ks_) under basal conditions and inhibition of L-type Ca^2+^ currents (I_CaL_) under cAMP-stimulated conditions (Nakamura et al., 2007). On the other hand, estradiol is demonstrated to potentiate QTc-prolonging effects of d,l-sotalol and to increase the susceptibility to d,l-sotalol-induced arrhythmias (Cheng et al., 2012), dominant probably due to upregulation of L-type Ca^2+^ channels (Yang et al., 2012, 2018) with greater dispersion in I_CaL_ density (Pham et al., 2002), reduced repolarization reserve, and enhanced Ca^2+^ overload (Sims et al., 2008). In transgenic long QT type 2 rabbits, estradiol promotes polymorphic ventricular tachyarrhythmias and sudden cardiac death although progesterone prevented them (Odening et al., 2012). Estradiol exerted the proarrhythmic effect by changing the arrhythmogenic substrate due to increased I_Ks_ and I_CaL_ currents, whereas progesterone, in contrast, exerted an antiarrhythmic effect by preventing early afterdepolarizations, likely due to an increase in SERCA2a and a decrease in the oscillatory I_CaL_ current (Odening et al., 2012). With regard to the opposing effects of estrogens and progesterone, slight changes in the progesterone:estrogen ratio may have profound consequences for cardiac repolarization and arrhythmia vulnerability as indicated by variable drug-induced QT prolongation in women during the menstrual cycle (Rodriguez et al., 2001).

In contrast to our findings, an earlier study of similar experimental design revealed no significant gender- and age-dependent differences in TdP incidence in a rabbit model of the acquired long QT syndrome (Johansson and Carlsson, 2001). Several factors probably contributed to this discrepancy: slightly lower age of rabbits in both young and adult groups (on average by 2 weeks), different anesthesia (methohexital and α-chloralose), and different I_Kr_ blocker (ibutilide). In contrast to dofetilide (used in our study), ibutilide in guinea pig cardiac myocytes at low concentrations increased the late inward current, and at high concentrations it increased the outward current (Lee et al., 1993). Consequently, ibutilide could, depending on the concentration, either enhance or depress the effect of K^+^ channel blockers on action potential duration.

### Effect of Hypercholesterolemia on TdP Inducibility

In our study, high cholesterol plasma levels did not influence susceptibility to TdP arrhythmias in the age-matched groups of the methoxamine-sensitized rabbit model. Levels of both non-HDL and HDL cholesterol were increased, whereas levels of triglycerides were comparable in both groups. Similarly, no association between HDL levels and ventricular repolarization indexes was found in subjects with primary hypercholesterolemia (Korantzopoulos et al., 2014). On the other hand, reconstituted HDL cholesterol shortened cardiac repolarization in isolated cardiomyocytes as well as in dyslipidemic patients (Den Ruijter et al., 2011) suggesting that decreased levels of HDL cholesterol (and not the elevated levels of total cholesterol) in patients with metabolic syndrome might contribute to QTc prolongation in these patients. In contrast to our results, pronounced neural and electrophysiological remodeling with nerve sprouting, prolonged action potential duration, longer QTc intervals, increased repolarization dispersion, and increased vulnerability to fibrillation were previously demonstrated in hypercholesterolemic rabbits (Liu et al., 2003). Both serum cholesterol levels and serum triglyceride levels were increased in these rabbits. Because, in our experiments, the cholesterol levels were increased to similar levels but triglycerides were not elevated, it is tempting to speculate that elevated plasma levels of triglycerides (but not of cholesterol) are necessary for inducing the proarrhythmic cardiac electrical remodeling.

### Autonomic Nervous System

The autonomic nervous system is an important modulator of cardiac electrophysiology and arrhythmogenesis (Shen and Zipes, 2014) and the α_1_-adrenergic stimulation is a prerequisite of arrhythmia induction in the rabbit model. Analysis of HRV at baseline revealed higher LF/HF ratios in rabbits susceptible to TdP arrhythmias, dominantly due to the lower HF component. These findings suggest a shift of the sympathovagal balance to sympathetic dominance due to the diminished vagal drive in TdP-susceptible rabbits, which is in line with putative protective effects of vagal stimulation against malignant ventricular arrhythmias (Brack et al., 2011). The protective effect of vagus nerve stimulation in isolated rabbit hearts was attributed to direct nitrergic action in the ventricle of post-ganglionic intracardiac nerves activated by vagus nerve (Brack et al., 2011).

### TdP Arrhythmia Prediction

To determine and predict the proarrhythmic risk, several electrophysiological parameters were analyzed and compared between groups of animals without any arrhythmia with TdP arrhythmias and with arrhythmias other than TdP (SEB, MEB, and bigeminy). In line with a number of previous studies (Thomsen et al., 2004), QTc intervals were not different between the groups, either at baseline or after challenge with I_Kr_ block. In contrast to the low predictive power of QTc interval prolongation, temporal STV of ventricular repolarization was repeatedly reported as a promising indicator of proarrhythmic risk in both experimental (Thomsen et al., 2004; Smoczynska et al., 2019) and clinical settings (Hinterseer et al., 2010; Orosz et al., 2015). In our rabbit model, STV in TdP-susceptible rabbits was indeed significantly increased in the presence of I_Kr_ block, and it was significantly different from the STV of rabbits without any arrhythmia. However, the interindividual variability in the group of TdP-susceptible rabbits was considerable, and the difference between the groups of TdP-susceptible rabbits and rabbits with other than TdP arrhythmias was not significant. It is questionable whether the insignificant difference in STV of TdP susceptible rabbits and rabbits with other than TdP arrhythmias was due to an insufficient predictive power of STV, or whether, in rabbits with other than TdP arrhythmias, a delayed development of TdP arrhythmias might occur (and these rabbits would, in reality, belong to the group of TdP-susceptible rabbits). The incidence of SEB and MEB was significantly higher in the group of TdP-susceptible rabbits, indicating their role in the genesis of TdP arrhythmias as suggested earlier (Verduyn et al., 1997). Both SEB and MEB showed rather high sensitivity but lower specificity. Anyway, it should be emphasized that both elevated STV and incidences of SEB and MEB only occurred in the presence of pharmacological challenge, IKr block, which considerably limits their potential clinical use.

Because analysis of entropy allows capturing the underlying dynamics of time series, it might possibly detect tiny irregularities of the ECG signal that reflect the differential susceptibility to arrhythmias even in baseline recordings. After testing various methods for analysis of time series, only methods based on entropy and sensitive to irregularities in amplitude revealed significant differences between baseline ECG records of arrhythmia susceptible and non-susceptible groups. Permutation entropy is a relatively simple time series complexity measure that is based on the Shannon information entropy and provides comparable results to other entropies, e.g., Kolmogorov–Sinai entropy (Unakafova, 2015). The standard permutation entropy analysis in our study, however, did not identify any significant differences between the groups, indicating that analyzing only the sample order but not the amplitude limits the power of the method as shown earlier (Cuesta-Frau, 2019). To overcome this amplitude-related limitation we have used the AAPE method as the most robust, simple, and effective method and, interestingly, it was able to distinguish the group without arrhythmias from other two arrhythmic groups. Furthermore, the entropy-based methods rely on moment sensory condition monitoring time series, and the time-varying measurement uncertainty cannot be properly managed (Zhang et al., 2013). To reduce the uncertainty within the diagnostic process, especially the time-varying measurement uncertainty, an information exergy-based method integrating multipoint and multimoment monitoring information was adopted from the field of structural damage diagnosis (Zhang et al., 2013), and the IEIN algorithm was developed (Zhang et al., 2014). The combination of AAPE and IEIN methods led to clustering of arrhythmia-free animals within arbitrary boundaries of the bottom-left quadrant. The other two groups of rabbits with TdP arrhythmias and rabbits with other than TdP arrhythmias, however, showed similar distribution patterns and could not be distinguished. In line with STV measurements, this perhaps suggests that TdP would eventually develop in the group of rabbits with other than TdP arrhythmias. Anyway, the data indicate that the entropy analysis of baseline ECG recordings (without any pharmacological challenge) could potentially stratify the arrhythmic risk and at least identify the low-risk individuals. To the best of our knowledge, the AAPE and EIEN approaches are so far never used for analysis of clinical ECG (either 12-lead or Holter), and perhaps, the concept could be extended to more arrhythmic phenotypes. From technical point of view, the application of entropy-based approaches to a clinical level should be easily possible; defining optimal patient groups, however, requires collaboration with experienced clinical cardiologists.

### Study Limitations

Statistically reliable analysis within individual age and cholesterol groups would require, with regard to the TdP incidence, rather high numbers of experimental animals (∼50 in each group). To reduce the number of animals used, we instead regrouped the animals according to arrhythmic endpoints, which allowed generalizing the predictive potential of the indicators across the experimental groups as required in clinical practice. Analysis of cardiac electrophysiological remodeling and proarrhythmic mechanisms on a cellular level would certainly add to understanding of the susceptibility or resistance to TdP arrhythmias in the rabbit model. It could also contribute to better distinction of the arrhythmic groups and elucidating whether the two arrhythmic groups show different TdP susceptibility due to intrinsic cellular (remodeling) differences or rather due to improper experimental design (e.g., a short duration of pharmacological challenge). Anyway, detailed analysis of cellular mechanisms of arrhythmia induction was beyond the scope of this study and will be addressed in future studies. In our study, only females were used, and gender differences cannot be excluded. LDL cholesterol levels were calculated with the Friedewald equation, which is routinely used in human medicine but was not verified for rabbits. Association between hypercholesterolemia and inflammation was not addressed in this study, and inflammatory markers were not measured. However, elevated CRP levels were described in a cholesterol-fed rabbit model previously (Yu et al., 2012), suggesting a good correlation with human pathology.

## Data Availability Statement

The raw data supporting the conclusions of this article will be made available by the authors, without undue reservation.

## Ethics Statement

The animal study was reviewed and approved by Committee for Experiments on Animals of the Charles University Faculty of Medicine in Pilsen and by the Ministry of Education, Youth, and Sports of the Czechia.

## Author Contributions

MŠ contributed to conception. MŠ and LN contributed to design of the study. LN, DJ, JŠ, AS, and JZ performed the experiments. DR performed cholesterol laboratory evaluation. JR and MK performed entropy analysis. DJ performed automatic and LN manual analysis of ECG. LN and JK performed the statistical analysis. LN and MŠ wrote the first draft of the manuscript. JR and MK wrote sections of the manuscript. All authors contributed to manuscript revision, read, and approved the submitted version.

## Conflict of Interest

The authors declare that the research was conducted in the absence of any commercial or financial relationships that could be construed as a potential conflict of interest.
